# Effectiveness of Mannheim’s Peritonitis Index in Patients With Peritonitis Secondary to Hollow Viscus Perforation in a Tertiary Care Hospital in Jharkhand, India

**DOI:** 10.7759/cureus.59631

**Published:** 2024-05-04

**Authors:** Kumar Gaurav, Kamlesh Kumar, Krishan Kumar, Anil K Kamal, Muklesh K Mehta, Hena Soy, Reema Bhagat

**Affiliations:** 1 General Surgery, Rajendra Institute of Medical Sciences, Ranchi, IND; 2 Surgery, Rajendra Institute of Medical Sciences, Ranchi, IND

**Keywords:** ranchi, tertiary care hospital, mortality and morbidity, organ failure, purulent, sepsis, peritonitis, perforation, mannheim peritonitis index

## Abstract

Introduction

Peritonitis refers to the inflammation of the peritoneum and peritoneal cavity. Causes of peritonitis can be bacterial (gastrointestinal or non-gastrointestinal), chemical, traumatic, or ischemic. Peritonitis can be localized or diffuse, acute or chronic. Peritonitis can be primary, secondary, or tertiary, according to the pathogenesis. Peritonitis developed secondary to hollow viscus perforation is a life-threatening condition and a common cause of emergency surgery in India. The Mannheim peritonitis index (MPI) is a simple scoring system that can accurately predict the outcome of peritonitis. This study aimed to evaluate the effectiveness of MPI in predicting mortality risk or prognosis in patients with peritonitis due to hollow viscus perforation.

Materials and methods

This observational cross-sectional study at the Department of General Surgery, Rajendra Institute of Medical Sciences, Ranchi, involved 111 patients with peritonitis due to hollow viscus perforation from December 2021 to March 2022. Detailed history, clinical examination, relevant blood tests, and radiological investigations established a diagnosis of perforation peritonitis, followed by a score assessment. Data were analyzed using SPSS software (IBM Corp., Armonk, NY, USA).

Results

Patients >50 years had higher mortality (i.e., 18/43) than patients <50 years (i.e., 13/68). Overall mortality was 31, which included one in low risk, 12 in intermediate risk, and 18 in the high-risk group. Mortality was lowest in the low-risk group (i.e., 1/30), highest in the high-risk group (i.e., 18/40), and 12/41 in the intermediate-risk group; the p-value was <0.05, which was highly significant. Mortality was higher in patients presenting after 24 hours, having organ failure, and non-colonic sepsis.

Conclusion

The MPI scoring system is simple, easy to calculate, cost-effective, precise, and effective in assessing mortality and morbidity risk in patients with peritonitis due to hollow viscus perforation. It can also guide further management strategies.

## Introduction

Peritonitis is defined as inflammation of the peritoneum and the surrounding cavity. Causes of peritonitis can be bacterial (gastrointestinal or non-gastrointestinal), chemical, traumatic, or ischemic. Peritonitis can be localized or diffuse, acute or chronic, or according to the pathology involved (primary, secondary, or tertiary peritonitis). Most commonly, in clinical settings, peritonitis categorization is based on whether it is localized or diffuse [1].

Peritonitis secondary to hollow viscus perforation is a frequent reason for emergency surgery in India, posing a life-threatening condition with very high (10-30%) mortality and morbidity despite advances in surgical techniques and the presence of resources and supportive care [2,3].

Treatment of peritonitis is primarily surgical, and in cases of doubt, especially patients who are previously healthy and those who present with postoperative peritonitis, early surgical intervention is always preferable.

In developing countries (such as India) with limited resources, early diagnosis and segmenting of patients with severe peritonitis could aid in determining those suitable for surgical intervention or intensive care approach. The causative factors and site of hollow viscus perforation vary between developed and developing nations [3].

Prognosis and outcomes depend on several factors (patient-related, disease-specific, and management). Many scoring systems, such as Acute Physiology and Chronic Health Evaluation II (APACHE II), Sepsis Severity Score (SSS), Boey score, and Mannheim peritonitis index (MPI) score, have been developed to assess prognosis and outcomes for patients with peritonitis [4]. Many of these scoring systems need several laboratory and radiological investigations, which may be unavailable in developing countries.

The MPI scoring system includes simple and readily available parameters and provides an easy method for assessing patients presenting with peritonitis secondary to hollow viscus perforation. Developed by Wacha and Linder, the MPI was based on data from 1243 peritonitis patients treated between 1963 and 1979, encompassing 17 potential risk factors, of which eight are currently used in MPI for mortality and morbidity prediction [5].

There are few studies on the effectiveness of the MPI scoring system in patients with peritonitis in India and other developing countries. This study aimed to assess the efficacy of the MPI scoring system in patients with peritonitis due to hollow viscus perforation at a tertiary care center in Jharkhand, predict mortality and morbidity, and determine optional treatment strategies for individual patients.

Aims and objectives

The study aimed to assess the effectiveness of MPI in predicting mortality or outcomes for patients with perforation peritonitis, determine the effects of various variables of the MPI scoring system, and predict prognosis accordingly.

## Materials and methods

This observational cross-sectional study was conducted at the Department of General Surgery, Rajendra Institute of Medical Sciences, Ranchi, a tertiary care hospital. The study included 111 patients with peritonitis due to hollow viscus perforation who underwent emergency surgery after consent from December 2021 to March 2022. The study received approval from the Institutional Ethics Committee of Rajendra Institute of Medical Sciences, Ranchi.

This study involved calculating the MPI score for each patient and assessing its effectiveness in predicting mortality or outcomes for patients with peritonitis caused by hollow viscus perforation. Additionally, the significance of each variable in predicting prognosis was investigated.

Inclusion criteria were age over 15 years and clinical suspicion of peritonitis caused by hollow viscus perforation, which was supported by investigations and confirmed after surgery.

Exclusion criteria were perforations due to trauma or firearm injury; peritonitis caused by vascular injury; significant comorbidities (such as uncontrolled diabetes mellitus, known chronic kidney disease, end-stage liver disease, and respiratory failure); death before operative procedures for peritonitis.

The patients received a detailed explanation of the study protocol and provided proper consent. The diagnosis of peritonitis secondary to hollow viscous perforation was based on a thorough history, clinical examination, and radiological assessments (chest X-ray PA-view in erect/abdomen in lateral decubitus position). Detailed histories of current illnesses and indications of long-term health conditions, such as cardiovascular, kidney, or liver disorders, were noted. Blood investigations were conducted, and relevant clinical information was recorded on admission. Stringent operative procedures were followed to address various etiologies of perforation peritonitis.

Based on palpatory findings, diffuse or localized peritonitis was noted, and based on the physical appearance of aspirate or exudate and intraoperative findings, the origin of sepsis (colonic or non-colonic) and the nature of the exudate (clear, purulent or fecal) was determined. Morbidity was evaluated based on postoperative complications, including surgical site infection, respiratory conditions (pneumonia or lung atelectasis or pleural effusion), cardiovascular compromise, renal failure, septic shock, and multi-organ failure.

The MPI score was determined by summing the individual variable scores obtained from risk variables found in the MPI, which were categorized based on indicated values using history, clinical examination, investigation values, and intra-operative findings. The cases were initially classified into three groups (based on Billing's classification): patients with scores below 21, between 21 and 29, and above 29 [6]. According to the weightage assigned to different variables in the MPI scoring system, the minimum possible score was 0 (if no risk factor), and the maximum score was 47 (in the presence of all risk factors) (Table 1).

**Table 1:** Mannheim Peritonitis Index Score *Organ failure: Renal failure (Oliguria <20 ml/hour, Sr. creatinine >1.6 mg/dl or B. urea >60 mg/dl or 167 mmol/L), Respiratory insufficiency (pO2 <50 mm Hg or pCO2 >50 mm Hg), Shock (SBP <90 mm of Hg or MAP <60 mm of Hg), Intestinal obstruction or paralytic ileus >24 hours or obstipation (complete mechanical obstruction). SBP - systolic blood pressure, MAP - mean arterial pressure, pO2 - partial pressure of oxygen, pCO2 - partial pressure of carbon dioxide.

S. No.	Risk factors		Score
1.	Age	>50 years	5
		<50 years	0
2.	Sex	Female	5
		Male	0
3.	Organ failure^*^	Present	7
		Absent	0
4.	Malignancy	Present	4
		Absent	0
5.	Peritonitis evolution period	>24 hours	4
		<24 hours	0
6.	Origin of sepsis	Non-colonic	4
		Colonic	0
7.	Extent of peritonitis	Diffuse	6
		Localized	0
8.	Exudate	Clear	0
		Purulent	6
		Fecal	12

The patient's condition was closely watched, including the occurrence of any complications and the decision to discharge based on improvements or in the unfortunate event of death. The duration between the initial diagnosis and occurrence of events (either death or hospital discharge) was documented. Data interpretation required assessing each variable in the scoring system as a separate predictor of morbidity or death and analyzing the system as a whole.

Statistical analysis

The data was encoded and inputted into an MS (Microsoft) Excel spreadsheet and then analyzed using SPSS (Statistical Package for Social Sciences) version 29.0 (IBM Corp., Armonk, NY, USA) software. The result was presented in tabular and graphical formats, depicting percentages. A data master sheet was generated for variables in the study. The paired Fisher Z-test was used to analyze the variables on a category scale across two or more groups to test the significance of the findings. A p-value less than 0.05 was considered statistically significant.

## Results

**Figure 1:**
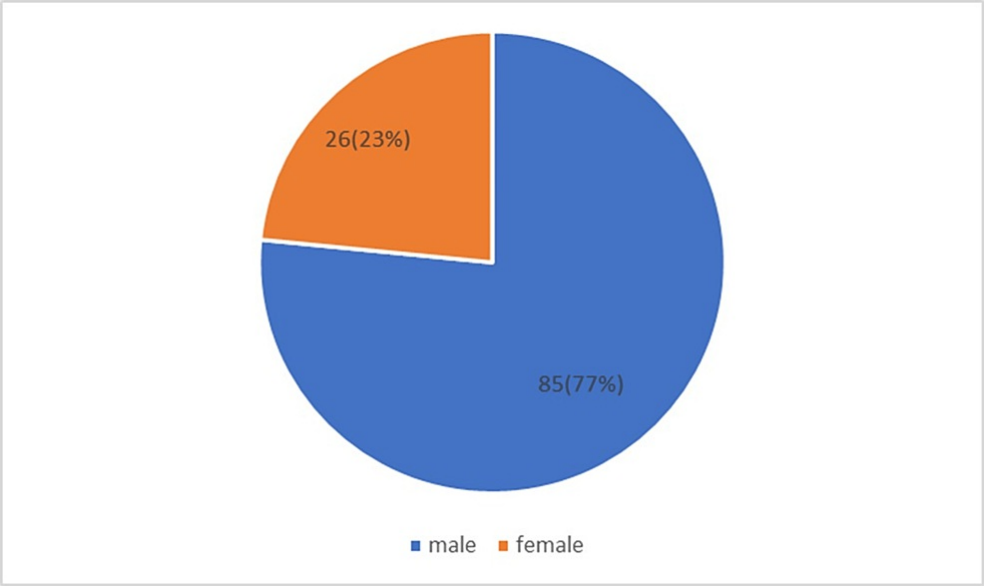
Prevalence among males and females.

There were 85 (77%) males and 26 (23%) females (3.27:1 ratio) (Figure 1). The age of patients ranged between 15-80 years.

**Figure 2:**
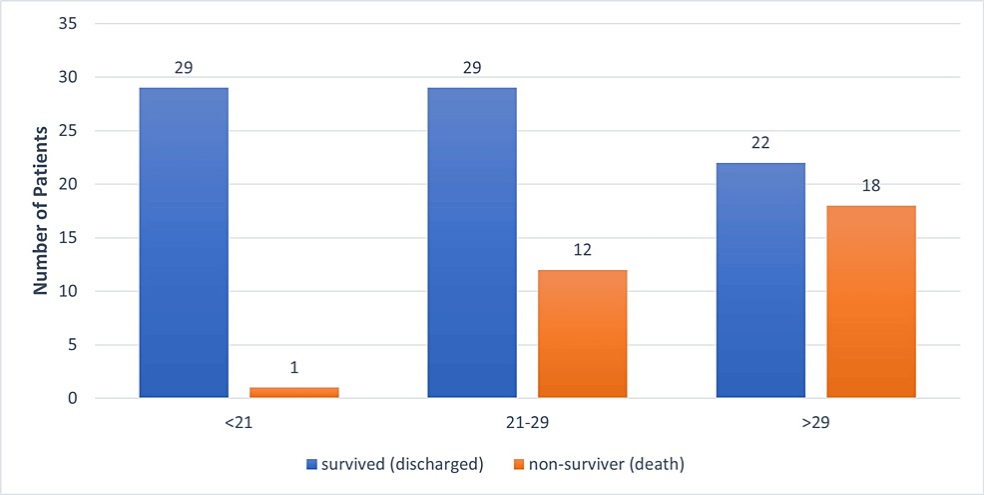
Outcome according to MPI score MPI - Mannheim's peritonitis index

In this study, overall mortality was 31 (27.92%), which included one in the low-risk, 12 in the intermediate-risk, and 18 in the high-risk group. Mortality was lowest in the low-risk group (i.e., 1/30 (3%)), highest in the high-risk group (i.e., 18/40 (45%)), and 12/41 (29%) in the intermediate-risk group; p-value was <0.05, which was highly significant (Table 2, Figure 2).

**Table 2:** Outcome according to MPI score (n=111) MPI - Mannheim's peritonitis score

Outcome	Survived (discharged)	Non-survived (death)	p-value
<21 (low-risk)	29 (97%)	1 (3%)	0.000598
21-29 (intermediate-risk)	29 (71%)	12 (29%)	
>29 (high-risk)	22 (55%)	18 (45%)	

In this study, patients >50 years had higher mortality (18/43 (42%)) than patients <50 years (i.e., 13/68 (19%)) (p = 0.0092). There was no discernible disparity in mortality among males (22/85 = 26%) and females (9/26 = 36%) (p = 0.385). However, females >50 years had very high mortality (i.e., 7/13 (54%)). Patients with organ failure had very high mortality (i.e., 28/47 (60%)) compared to patients with no organ failure at presentation (p <0.00001). Almost all mortality was found in patients who presented with >24 hours evolution time (i.e., 31/100 (31%)) (p = 0.1005). Most patients had a non-colonic origin of sepsis 107/111 (96%) with mortality of 29/107 (27%) (p = 0.316). Most patients presented with generalized diffuse peritonitis (106/111) with a mortality of 31/106 (29%), and there was no mortality in patients who presented with localized peritonitis (0/5) (p = 0.507). Patients having fecal contamination had very high mortality (i.e., 2/3 (67%)) compared to patients having purulent exudate (i.e., 26/83 (31%)) and clear exudate (i.e., 3/25 (12%)) (p = 0.053) (Table 3).

**Table 3:** Outcome according to different variables of MPI score and p-value MPI - Mannheim's peritonitis index

Risk factors		Survived	Non-survived	p-value
Age	<50 years	55 (81%)	13 (19%)	0.0092
	>50 years	25 (58%)	18 (42%)	
Sex	Male	63 (74%)	22 (26%)	0.385
	Female	17 (65%)	9 (35%)	
Organ failure	Present	19 (40%)	28 (60%)	<0.00001
	Absent	61 (95%)	3 (5%)	
Malignancy	Present	1 (50%)	1 (50%)	0.482
	Absent	79 (72%)	30 (28%)	
Peritonitis evolution period	<24 hours	11 (100%)	0 (0%)	0.1005
	>24 hours	69 (69%)	31(31%)	
Origin of sepsis	Non-colonic	78 (73%)	29 (27%)	0.316
	Colonic	2 (50%)	2 (50%)	
Extent of peritonitis	Localized	5 (100%)	0 (0%)	0.507
	Diffuse	75 (71%)	31 (29%)	
Exudate	Clear	22 (88%)	3 (12%)	0.053
	Purulent	57 (69%)	26 (31%)	
	Fecal	1 (33%)	2 (67%)	

**Figure 3:**
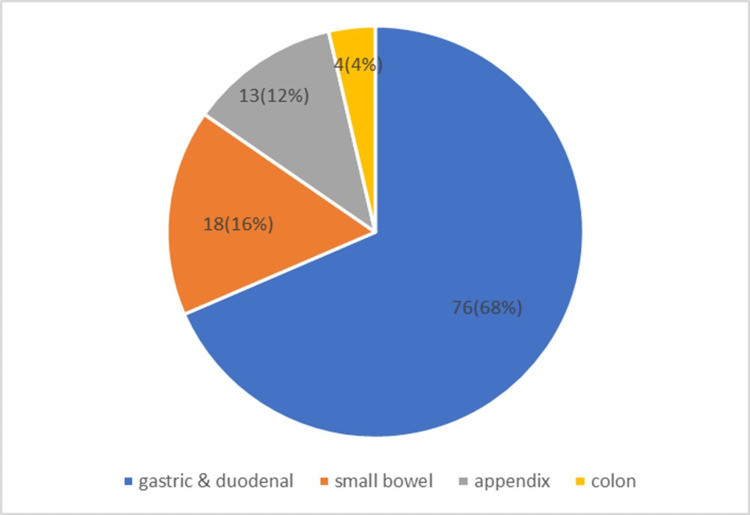
Site of perforation

The most common site of perforation was gastric or duodenal (i.e., 76 (68%)), followed by small bowel (i.e., 18 (16%)), appendix (i.e., 13 (12%)), and colon (i.e., 4 (4%)) (Figure 3).

**Figure 4:**
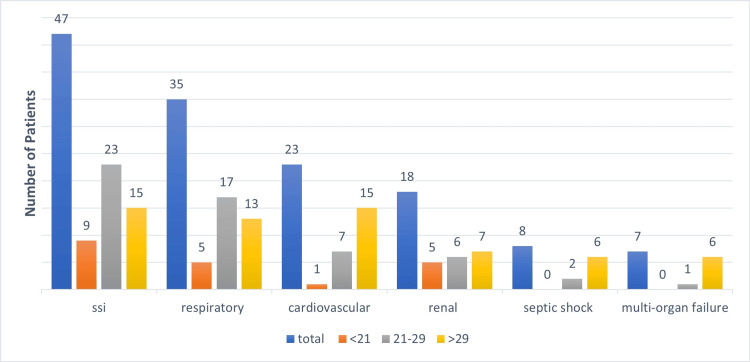
Post-operative complication and MPI score SSI - Surgical site infection, MPI - Mannheim's peritonitis index

The most common postoperative complication or morbidity was surgical site infection (SSI) (i.e., 47/111 (42%)), followed by respiratory failure (i.e., 35/111 (31.5%)), cardiovascular compromise (i.e., 23/111 (21%)), renal failure (i.e., 18/111 (16%)), septic shock (i.e., 8/111 (7%)), and multi-organ failure (i.e., 7/111 (6%)). SSI was the most common complication in patients with MPI scores <21; respiratory failure and SSI were more common in patients with MPI scores 21-29, and patients with MPI scores >29 were more prone to cardiovascular complications. Multi-organ failure, renal failure, and septic shock were found mostly among patients with MPI scores >29 (Figure 4).

**Figure 5:**
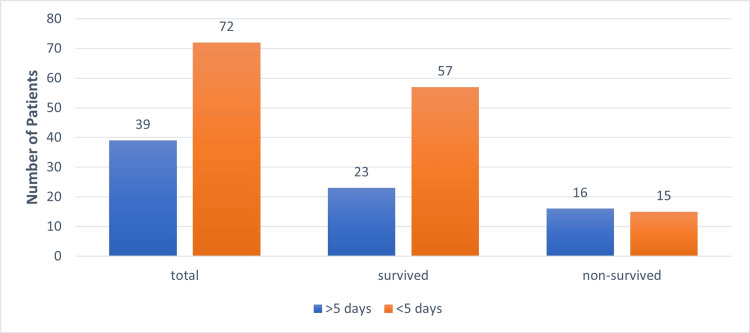
ICU stay and mortality ICU - Intensive care unit

In our study, 39/111 (35%) patients needed >5 days of intensive care unit (ICU) stay and exhibited higher mortality (i.e., 16/39 (41%)) than patients who needed <5 days ICU stay (15/72 (21%)) (Figure 5).

## Discussion

Peritonitis developed due to hollow viscus perforation is one of the most common causes of surgical emergencies. Categorization of all patients presenting to emergency with perforation peritonitis would help in deciding patient management strategies because, despite much advancement in the medical and surgical field, mortality in perforation peritonitis patients remains very high. MPI scoring system uses history, clinical examination findings, initial investigation, aspirate, or intra-op findings.

In our study, patients >50 years had higher mortality (18/43 (42%)) than patients <50 years (i.e., 13/68 (19%)), and a p-value of 0.0092 showed a positive significance of more mortality with increasing age >50 years. This result is consistent with the findings of Sharma et al. [7].

There were more males in our study (male:female = 3.26:1), and there was no significant relation with mortality (p-value was 0.385), which is consistent with studies by Mathur and Sharma [8] and Huttunen et al. [9] and in contrast to the study by Sharma et al. [7] (where females had higher mortality than males). However, our study showed higher mortality (54%) among females >50 years.

In the present study, patients with organ failure at presentation had very high mortality (i.e., 28/47 (60%)), with a positive significance with mortality (p-value <0.00001), which is similar to studies by Sharma et al. [7].

In our study, risk factors, such as malignancy (p = 0.482), peritonitis evolution period (p = 0.1005), origin of sepsis (p = 0.316), and extent of peritonitis (p = 0.507) had no positive significance, in contrast to studies by Sharma et al. [7] and Bohnen et al. [10]. However, in our study, almost all mortality was found in patients who presented with >24 hours evolution time (i.e., 31/100 (31%)). Most patients had a non-colonic origin of sepsis (107/111 (96%)) with a mortality of 29/107 (27%). Most patients presented with generalized diffuse peritonitis (106/111) with a mortality of 31/106 (29%), and there was no mortality in patients with localized peritonitis (0/5).

Patients having fecal contamination had higher mortality (67%) than patients having purulent and clear exudate, although it is not statistically significant (p-value = 0.053), but mortality in fecal contamination was similar to the findings of Sharma et al. [7].

The most common perforation site was gastric or duodenal (i.e., 76 (68%)), followed by small bowel (i.e., 18 (16%)), appendix (i.e., 13 (12%)), and colon (i.e., 4 (4%)). This finding is consistent with the study by Muralidhar et al. [11] and in contrast with Ramteke et al. [12] (which showed the most common perforation site as the appendix followed by duodenum).

Overall mortality was 31 (28%). Mortality among the low-risk group (MPI <21) was lowest (i.e., 1/30 (3%)) and highest among the high-risk group (MPI >29) (i.e., 18/40 (45%)); mortality was 12/41 (29%) in the intermediate-risk group (MPI 21-29). There was positive significance (p < 0.05) with mortality, consistent with the studies by Barrera et al. [13], Sharma et al. [7], and Ramteke et al. [12] (i.e., mortality increases with an increase in MPI score).

Our study showed that patients with higher MPI scores were more prone to various complications or morbidity, such as surgical site infection, respiratory insufficiency, cardiovascular compromise, renal failure, systemic inflammatory response syndrome, shock, and multi-organ failure, which is in agreement with the study by Ramteke et al. [12] and Budzyński et al. [14]. Surgical site infection was the most common complication in patients with MPI scores <21; respiratory failure and SSI were more common in patients with MPI scores 21-29, and patients with MPI scores >29 were more prone to cardiovascular complications. Multi-organ failure, renal failure, and septic shock were most common in patients with MPI scores >29. Obese individuals experienced a higher incidence of complications, including surgical site infections and respiratory insufficiency. Additionally, they had prolonged stays in the intensive care unit and the hospital.

Results showed that 72% (80/111) of patients got discharged and 28% (31/111) exhibited mortality; the majority of mortality was in patients with MPI >29, and mortality increased in patients with higher MPI scores, similar to other studies [5,6,15-17].

The limitation of this scoring system is that it uses both preoperative and intraoperative findings, precluding preoperative assessment of prognosis; the origin of sepsis, the nature of the exudate, and the intraoperative findings significantly affected the fate of patients. And peritonitis caused by factors other than perforation of hollow viscus, such as spontaneous peritonitis, tuberculous peritonitis, post-operative peritonitis, peritonitis following blunt trauma abdomen, and so on were not covered in this study.

## Conclusions

The variables of the scoring system that significantly contributed to predicting patient prognosis were age, peritonitis evolution period, organ failure, and the presence of purulent and fecal exudates. The delayed arrival of patients to our hospital may have influenced our findings, leading to an increased reporting of mortality.

This study demonstrates that the MPI scoring system is simple, easy to calculate, cost-effective, precise, and effective in assessing mortality and morbidity in patients with peritonitis due to hollow viscus perforation. The MPI scoring system can guide patient management after definitive procedures, such as critical care-based treatment and higher antibiotic usage, for improving patient prognosis.
